# The Insect Ortholog of the Human Orphan Cytokine Receptor CRLF3 Is a Neuroprotective Erythropoietin Receptor

**DOI:** 10.3389/fnmol.2017.00223

**Published:** 2017-07-14

**Authors:** Nina Hahn, Debbra Y. Knorr, Johannes Liebig, Liane Wüstefeld, Karsten Peters, Marita Büscher, Gregor Bucher, Hannelore Ehrenreich, Ralf Heinrich

**Affiliations:** ^1^Department of Cellular Neurobiology, Institute for Zoology, Georg-August-University Goettingen Goettingen, Germany; ^2^Clinical Neuroscience, Max Planck Institute of Experimental Medicine Goettingen, Germany; ^3^DFG Center for Nanoscale Microscopy and Molecular Physiology of the Brain (CNMPB) Goettingen, Germany; ^4^Department of Evolutionary Developmental Biology, Institute for Zoology, Georg-August-University Goettingen Goettingen, Germany

**Keywords:** erythropoietin, non-erythropoietic splice variant, neuroprotection, cytokine receptor, primary neuronal cell culture, RNA interference, *Tribolium castaneum*

## Abstract

The cytokine erythropoietin (Epo) mediates various cell homeostatic responses to environmental challenges and pathological insults. While stimulation of vertebrate erythrocyte production is mediated by homodimeric “classical” Epo receptors, alternative receptors are involved in neuroprotection. However, their identity remains enigmatic due to complex cytokine ligand and receptor interactions and conflicting experimental results. Besides the classical Epo receptor, the family of type I cytokine receptors also includes the poorly characterized orphan cytokine receptor-like factor 3 (CRLF3) present in vertebrates including human and various insect species. By making use of the more simple genetic makeup of insect model systems, we studied whether CRLF3 is a neuroprotective Epo receptor in animals. We identified a single ortholog of CRLF3 in the beetle *Tribolium castaneum*, and established protocols for primary neuronal cell cultures from *Tribolium* brains and efficient *in vitro* RNA interference. Recombinant human Epo as well as the non-erythropoietic Epo splice variant EV-3 increased the survival of serum-deprived brain neurons, confirming the previously described neuroprotective effect of Epo in insects. Moreover, Epo completely prevented hypoxia-induced apoptotic cell death of primary neuronal cultures. Knockdown of CRLF3 expression by RNA interference with two different double stranded RNA (dsRNA) fragments abolished the neuroprotective effect of Epo, indicating that CRLF3 is a crucial component of the insect Epo-responsive receptor. This suggests that a common urbilaterian ancestor of the orphan human and insect cytokine receptor CRLF3 served as a neuroprotective receptor for an Epo-like cytokine. Our work also suggests that vertebrate CRLF3, like its insect ortholog, might represent a tissue protection-mediating receptor.

## Introduction

Erythropoietin (Epo) is a positive regulator of vertebrate erythropoiesis. Circulating hormonal Epo, predominantly released by the kidney in adult mammals, stimulates homodimeric Epo receptors (“classical EpoR”) on Epo-responsive erythroid progenitor cells in the bone marrow that initiate anti-apoptotic mechanisms leading to increased numbers of red blood cells (Jelkmann, 1992). Epo is also synthesized and released as a para- or autocrine signal in various tissues that initiates adaptive cellular responses to injury and physiological challenges in order to support cell survival, maintenance of functions and regeneration (Noguchi et al., 2008; Sargin et al., 2010; Chateauvieux et al., 2011). In the nervous system, Epo is produced by glia, neurons and endothelial cells. Epo plays important roles during mammalian brain development (Alnaeeli et al., 2012), acts as a protectant that secures neuronal survival and functionality under ischemic, cytotoxic and otherwise deleterious conditions (Brines and Cerami, 2005; Arcasoy, 2008; Sargin et al., 2010), supports regeneration (Kretz et al., 2005; King et al., 2007) and enhances cognitive performance in healthy and diseased brains (Sargin et al., 2011; Kästner et al., 2012). Although expression of classical EpoR in the brain has been demonstrated by numerous studies, Epo receptors mediating neuroprotection seem to be different from the classical homodimeric EpoR involved in erythropoiesis (Leist et al., 2004; Brines and Cerami, 2005). Several Epo-like molecules have been demonstrated to lack erythropoietic functions while retaining full neuroprotective activity compared to normal Epo. Such tissue-specific agonists include the derivatives carbamylated Epo and asialoerythropoietin (Erbayraktar et al., 2003; Leist et al., 2004), mutant Epo with serine to leucine mutation at position 104 (Gan et al., 2012) and the human Epo splice variant EV-3 with deleted third exon (Bonnas, 2012). The nature of the neuroprotective Epo receptor is currently debated. As non-erythropoietic candidate receptors, common β chain receptor/classical EpoR heteromers expressed in the nervous system (Brines and Cerami, 2005; Collino et al., 2015) and bone tissue (Duedal Rölfing et al., 2014) and leukemia inhibitory factor LIFR/DC130 expressed in neuronal and other cell lines (Bonnas, 2012) are discussed. However, even within a particular tissue such as the nervous system, the expression of Epo receptors may depend on cell type, developmental stage, physiological condition and actual as well as previous exposure to challenging stimuli (Sinor and Greenberg, 2000; Brines and Cerami, 2005; Shein et al., 2005; Um et al., 2007; Sanchez et al., 2009). Cells may flexibly respond to different physiological challenges by expressing different Epo receptors that activate particular adaptive cellular responses.

Genes for Epo and classical EpoR are present in all major vertebrate groups including mammals, amphibia and fish (Ostrowski et al., 2011), suggesting that Epo/EpoR signaling already evolved when the vertebrate lineage emerged. Based on protein sequence similarity, which is already quite low between mammals and fish, no orthologs of Epo and EpoR have so far been identified in invertebrate species. Nevertheless, Epo-mediated neuroprotection and neuroregeneration have been described in grasshoppers and locusts (Ostrowski et al., 2011; Miljus et al., 2014). In these studies recombinant human Epo rescued primary cultured brain neurons from hypoxia-induced apoptotic cell death by activating Janus kinase/signal transducers and activators of transcription (JAK/STAT) transduction pathways (Miljus et al., 2014). In addition, locust brain neurons were also protected by the non-erythropoietic human splice variant EV-3 (Miljus et al., 2017; characterization of EV-3 by Bonnas et al., 2017), suggesting that insect and mammalian neuroprotective Epo receptors share common structures that allow activation by both Epo and EV-3. These findings support the hypothesis of a pre-vertebrate evolution of Epo-like signaling in neuroprotection (Brines and Cerami, 2005).

Epo belongs to the group of type I helical cytokines that, in spite of low primary sequence similarity, share spatial similarity emerging from a characteristic four-helix bundle structure (Boulay et al., 2003). Their corresponding receptors (type I cytokine receptors) contain a conserved 200 amino acid “cytokine receptor homology domain” required for ligand binding and some conserved intracellular motifs that allow the activation of JAK/STAT transduction pathways (Boulay et al., 2003; Liongue and Ward, 2007). Cytokine type I receptors typically associate to homo- or heteromeric receptor complexes that may bind several cytokine ligands.

Classical EpoR belongs to group 1 of cytokine type I receptors, which also includes thrombopoietin receptor, prolactin receptor, growth hormone receptor and the orphan cytokine receptor-like factor 3 (CRLF3) of unknown function. CRLF3 (synonyms: CYTOR4, CREME9, p48.2, p48.6 (sequences may deviate by few amino acids at the 5′ end) has been shown to activate STAT3, cause cell cycle arrest in a human embryonic kidney cell line (Yang et al., 2009) and may regulate neuronal morphology and synaptic vesicle formation (Hashimoto et al., 2012). *Crlf3* is located on human chromosome 17 and is expressed in most normal human tissues including the nervous system. Variations of *crlf3* sequences have been associated with amyotrophic lateral sclerosis (ALS; Cirulli et al., 2015) and increased CRLF3 content has been detected in various tumor cell lines and freshly isolated tumors (Dang et al., 2006; Yang et al., 2009). *Crlf3* is well conserved which allowed the identification of orthologs in various mammals, fish, primitive chordates and insects, such as the beetle *Tribolium castaneum* and the cricket *Gryllus bimaculatus* but not in the fly *Drosophila melanogaster* (Wyder et al., 2007). Interestingly, we were able to show a neuroprotective effect of Epo on *T. castaneum* (this study) and *G. bimaculatus* neural cells but failed to do so in *D. melanogaster* (unpublished results) which led us to hypothesize that CRLF3 might be mediating this effect.

The red flour beetle *T. castaneum* has a fully sequenced genome and is amenable to double stranded RNA (dsRNA)-mediated systemic RNAi (Bucher et al., 2002). In order to explore the possibility that beetle CRLF3 (*Tc-*CRLF3) plays a role in Epo-mediated neuroprotection, we established primary neuronal cell cultures from *T. castaneum* brains and effective soaking RNA interference by dsRNA added to the culture medium. We demonstrate that knockdown of *Tc*-CRLF3 expression abolishes the protective effect of Epo on hypoxia-exposed *T. castaneum* brain neurons.

## Materials and Methods

### Plasmids

A 427 bp long fragment of the red fluorescent protein (dsRed) of the mushroom anemona *Discosoma spec*. was cloned into pCRII vector by the Bucher Lab. Additionally, 300 bp of Rpt3 were cloned into pJet 1.2 (Thermo Fisher Scientific, Waltham, MA, USA; Ulrich et al., 2015). For RNAi experiments targeting *Tc*-CRLF3, primer pairs for two different fragments targeting *au3.g2971.t1 (Tc000209)* were designed (Table 1). The fragment 1 covers 638 bp whereas the fragment 2 is smaller and consists of 527 bp (sequences shown in Supplementary Figure S1). The fragments were cloned into the pCRII vector using the TA Cloning^®^ Kit (Invitrogen, Life Technologies, Darmstadt, Germany). Subsequently, the plasmids were transformed into DH5α cells and finally purified with the NucleoSpin^®^ Plasmid Kit (Macherey-Nagel, Düren, Germany).

**Table 1:** Sequences of primers.

Primer	Sequence
*crlf3* fragment 1 forward	5′ GGGTGATAGGAACGAAGTGGTGGA 3′
*crlf3* fragment 1 reverse	5′ GACGTCATATCTGAGAAACACTTAG 3′
*crlf3* fragment 2 forward	5′ CGATTGTTATGTGGGCGCAGAGAC 3′
*crlf3* fragment 2 reverse	5′ GAGTCAGTATTGATACGTGTAACA 3′
M13F (−20)	5′ GTAAAACGACGGCCAGT 3′
T7-M13R (−17)	5′ TAATACGACTCATAGGCAGGAAACAGCTATGAC 3′
T7-pJet1.2 forward	5′ TAATACGACTCACTATAGGCGACTCACTATAGGGAGAGC 3′
T7-pJet1.2 reverse	5′ TAATACGACTCACTATAGGAAGAACATCGATTTTCCATGGCAG 3′

### Preparation of dsRNA

Template DNA was prepared by PCR using M13F and M13R primers with a T7 RNA polymerase promoter sequence attached to the latter (Table 1). *In vitro* transcription of dsRNA was performed with the MEGAscript^®^ T7 Transcription Kit following the user manual (Life Technologies, Darmstadt, Germany). The resulting RNA pellet was resuspended in injection buffer (1.4 mM NaCl, 0.07 mM Na_2_HPO_4_, 0.03 mM KH_2_PO_4_, 4 mM KCl). In order to anneal both strands of the RNA, the mix was incubated at 94°C for 5 min and cooled down slowly in a heatblock. Before use in cell culture experiments it was sterile filtered (Millex^®^-HV, 0.45 μm, hydrophilic durapore, Millipore, Darmstadt, Germany).

### Sequence Comparison

Information about *Tribolium* gene sequence and structure was gained from http://bioinf.uni-greifswald.de/gb2/gbrowse/tribolium4/ working with the augustus3 prediction. Sequences were identified with Basic Local Alignment Search Tool (BLAST; Altschul et al., 1990) using the matrix BLOSUM62 with standard settings and the mode tblastn. Sequence information of other species was retrieved with BLAST at NCBI data bases (Altschul et al., 1990).

Geneious^®^ (Biomatter Ltd., Auckland, New Zealand) was utilized for alignments using the algorithm ClustalW with standard settings. To identify orthologs of *Tribolium castaneum* CRLF3, a phylogenetic tree was built with the bootstrap resembling method and standard settings as follows: The initial protein sequence alignment was created with the first three hits of *Tribolium castaneum* and *Drosophila melanogaster* and the first four hits of *Mus musculus*. Domains with high quality of alignments were extracted and concatenated for tree building.

Phylogenetic analysis of the CRLF3 protein was also performed with Geneious^®^. Orthologous amino acid sequences were aligned with ClustalW algorithm with standard settings. The tree was calculated using the Geneious^®^ tree builder with Neighbor-Joining method and bootstrap resampling with default settings. The analysis includes CRLF3 orthologs of the human CRLF3 protein identified by NCBI BLAST. They belong to *Homo sapiens* (GenBank accession number NP_057070.3), *Mus musculus* (NP_061246.1), *Danio rerio* (NP_001017817.2), *Xenopus laevis* (NP_001080600.1), *Branchiostoma floridae* (XP_002607148.1), *Ciona intestinalis* (NP_001107600.1) and *Tribolium castaneum* (XP_008190449.1).

### Animals

Studies were performed with red flour beetles *Tribolium castaneum* (wild-type strain San Bernadino). The beetles were bred in plastic boxes filled with a mixture of full grain flour (Wheat flour type 405; Rosenmühle GmbH, Landshut, Germany) and yeast at 27°C and 40% air humidity. The study was conducted exclusively with insects. Studies on insects do not require special permit. All experiments comply with the German laws for animal welfare (“Deutsches Tierschutzgesetz”).

### Primary Cultures of *T. Castaneum* Brain Cells

Primary cultures were established from brains of late pupae. Brains were extracted from head capsules and collected in 2 ml sterile growth medium (Leibovitz’s L-15 (Gibco, Karlsruhe, Germany) supplemented with 0.5% gentamicin (BioReagent, Munich, Germany)). All brains used in one experiment (20 brains per treatment group) were pooled at this stage. After three exchanges of the medium, the brains were exposed to a mixture of collagenase and dispase (each 2 mg/ml; Sigma-Aldrich, Munich, Germany) for 45 min at 27°C to digest extracellular matrix proteins. Enzymatic activity was terminated by several washes in Hanks’ Balanced Salt Solution (Gibco, Karlsruhe, Germany). Brain tissue was passed several times through the tip of a 100 μl pipette to dissociate the brain cells. The suspension was centrifuged with 3000 *g* for 1 min and the pellet containing the cells was resuspended in L-15 medium with 0.5% gentamicin (100 μl per treatment group). The dissociated cells were equally allocated onto concanavalin A-coated (Sigma-Aldrich, Munich, Germany) round cover slips (Type DKR0; Hartenstein; Würzburg, Germany; Ø 10 mm; 100 μl per cover slip) that were placed in sterile plastic culture dishes (Corning Inc., New York, NY, USA; Ø 35 mm). After 2 h the culture dishes were filled to a volume of 2 ml with L-15/0.5% gentamicin ± supplements as specified below. Cultures were maintained in an incubator (27°C; humidified atmosphere) and medium was replaced every 2 days.

### Immunocytochemistry

Primary cell cultures were fixed with 4% paraformaldehyde dissolved in 0.1 M phosphate buffer for 15 min at room temperature. Cells were washed with phosphate buffered saline (PBS) and PBS with 0.1% Triton-X-100 (Sigma-Aldrich, Munich, Germany; PBST), each three times for 5 min. Cells were incubated for 1 h with blocking solution containing 2% normal goat serum (GE Healthcare, Munich, Germany) and 0.25% bovine serum albumin (MP Biochemicals, Heidelberg, Germany) dissolved in PBST. Primary antisera were incubated over night at 4°C. To label neuronal membranes anti-horseradish peroxidase (anti-HRP from rabbit, Sigma Aldrich) was applied in 1:500 dilution in blocking buffer. To label the activated form of caspase-3, anti-cleaved caspase-3 serum from rabbit (Calbiochem, Merck, UK) was applied in 1:300 dilution in blocking buffer. After several washes in PBS, cells were incubated with 1:200 diluted Alexa 488-coupled anti-rabbit secondary antibody (developed in goat, Molecular Probes, Thermo Fisher Scientific, Waltham, MA, USA) for 2 h at room temperature. In some experiments, DAPI (Sigma-Aldrich, Munich, Germany; 1:1000) was applied with the secondary antibody. After washing away excess antibody, cover slips with labeled cells were mounted with 1,4-diazobicyclo[2.2.2]octane (DABCO, Roth, Karlsruhe, Germany) on microscopic slides. Edges of cover slips were sealed with transparent nail polish and stainings were analyzed by fluorescence microscopy.

### Protective Effect of rhEpo and EV-3 on *T. Castaneum* Brain Cells *In Vitro*

Four-day-old primary cultures were maintained in L-15 medium supplemented with 0.5% gentamicin. Particular cultures were incubated with different concentrations (0.32 ng/ml, 0.8 ng/ml, 3.2 ng/ml, 8 ng/ml, 32 ng/ml) of recombinant human Epo (rhEpo, NeoRecormon, Roche, Welwyn Garden City, UK) or (0.42 ng/ml, 0.84 ng/ml) of the human non-erythropoietic Epo splice variant EV-3 (IBA GmbH, Göttingen, Germany) for another 3 days. Cell cultures were fixed in paraformaldehyde (4%, 30 min), labeled with DAPI (Sigma-Aldrich, Munich, Germany; 1:1000) and mounted with DABCO (Roth, Karlsruhe, Germany) on microscopic slides to quantify cellular survival (see below).

### Soaking RNA Interference

Primary cultures of *T. castaneum* brains were maintained in L-15 medium supplemented with 0.5% gentamicin for 4 days. Each experiment, using the cultured cells from the same population of 80 brains, contained four different treatment groups, one control, incubated without any supplement throughout the entire period and three cultures that were supplemented with 10 ng/μl dsRNA to suppress the expression of dsRed (a protein not naturally expressed by the cells), *Tc-*CRLF3 (*T. castaneum* ortholog of CRLF3) and (Regulatory particle triple-A ATPase 3 (Rpt3), a protein of the proteasome that is essential for cellular survival), respectively (for dsRNA sequences see Supplementary Figure S1). After 4 days *in vitro*, cell cultures were fixed and labeled with DAPI as described above.

### Survival in Hypoxia

Primary cultures of *T. castaneum* brains were maintained in L-15 medium supplemented with 0.5% gentamicin for 5 days. Each experiment, using the cultured cells from the same population of 100 brains, contained five different treatment groups: (1) untreated control maintained in normoxic condition; (2) untreated control exposed to hypoxia; (3) rhEpo-treated culture exposed to hypoxia; (4) rhEpo-treated culture exposed to hypoxia after RNAi-mediated suppression of *Tc-*CRLF3 expression; and (5) hypoxia-exposed culture after RNAi mediated suppression of *Tc-*CRLF3 expression. dsRNA (final concentration 10 ng/μl) was present in the respective cultures throughout the entire experimental period. To minimize the risk for off-target effects, two dsRNA constructs (see Supplementary Figure S1) that target non-overlapping sequences were used to interfere with *Tc-*CRLF3 expression. rhEpo (0.8 ng/ml) was added to the respective cultures 12 h before the hypoxic period. Hypoxia was generated by floating an airproof chamber (22.5 × 4.5 × 4 cm, workshop of our institute) with nitrogen (N_2_). Hypoxia (O_2_ level ≤ 2%) was monitored with an oxygen analyzer (Greisinger GOX 100, Conrad Electronics, Hirschau, Germany) and maintained for 36 h. Afterwards, cultures were reoxygenated, maintained for 12 h in normoxia, and subsequently fixed and labeled as described above.

### Data Analysis

Assessment of cellular survival was based on chromatin structure revealed by fluorescent labeling with DAPI (Gocht et al., 2009). Using a fluorescence microscope (Zeiss Axioskop, Oberkochen, Germany) with 63× oil objective, equipped with a CCD camera (Spot RT3, Visitron, Puchheim, Germany), two continuous rows of non-overlapping photographs were taken (typically ~120 per culture), that extended over the entire cover slip to the right and the left of the center. Numbers of nuclei from alive and dead cells (at the time of fixation) were manually counted with the freeware software Fiji and its cell counter plugin (ImageJ by NIH). Relative numbers of alive cells were calculated for each culture. Within each experiment (that included cultured neurons from the brains of the same individuals) the values were normalized to survival in the untreated control culture (≙100%). Differences in data population distributions were probed with the non-parametric Kruskal-Wallis test, and the Mann-Whitney *U* test for unmatched samples was used to determine the differences between two groups (statistics performed with OriginPro 8.5, OriginLab Corporation). To adjust the false discovery rate, *P* values were corrected with the Benjamini-Hochberg procedure (Benjamini and Hochberg, 1995; Groppe et al., 2011).

## Results

### Sequence Analysis

The *T. castaneum* gene prediction *au3.g2971.t1 (Tc000209, Tc-crlf3*) is an ortholog of the human *crlf3*. It is located on chromosome linkage group 2 (ChLG2) at position 14179075–14181074. The size of the mRNA is 1803 bp and the coding sequence, distributed over five exons, comprises 1272 bp. Conserved domains of the 424 amino acid containing *Tc-*CRLF3 protein include a fibronectin type III domain, a cytokine receptor motif and interdomain contacts (Supplementary Figure S2).

**Figure 1:**
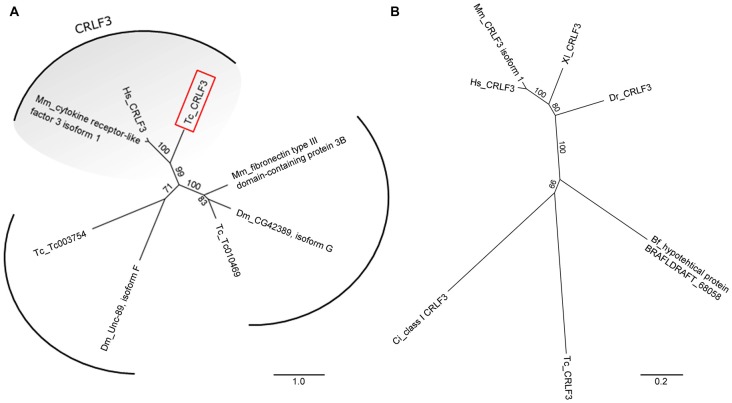
Unrooted phylogenetic trees showing the relationship of cytokine receptor-like factor 3 (CRLF3) proteins. **(A)** Phylogenetic tree originating from Basic Local Alignment Search Tool (BLAST) with the human CRLF3 protein sequence (Hs_CRLF3). Protein sequences from *Tribolium castaneum* (Tc), *Drosophila melanogaster* (Dm) and *Mus musculus* (Mm) were considered for the alignment. *Tribolium* Tc-CRLF3 (red box) is an ortholog of the human CRLF3 since it belongs to the CRLF3 group. Paralogs are not present in *Tribolium*. **(B)** CRLF3 orthologs of *Homo sapiens* (Hs), *Mus musculus* (Mm), *Danio rerio* (Dr), *Xenopus laevis* (Xl), *Branchiostoma floridae* (Bf), *Ciona intestinalis* (Ci) and *Tribolium castaneum* (Tc) were compared with respect to phylogenetic relationship. Orthologs of mammalian as well as of vertebrate CRLF3 separate in well supported branches. Table 2 summarizes the identity of the respective proteins.

A phylogenetic tree revealed clear orthology of human, mouse and *Tribolium* CRLF3 proteins (Figure 1A). Importantly, no further paralogs were detected in *Tribolium* which could act redundantly and possibly rescue RNAi-induced phenotypes (Figure 1A).

CRLF3 orthologs are present in mammals, amphibia, fish, cephalochordates, tunicates and insects. Among insects, *crlf3* genes were identified in the beetle *T. castaneum* and the cricket *Gryllus bimaculatus* (^1^Gene: *GB-isotig00932*) but not in fruit flies, mosquitoes and honey bees (Wyder et al., 2007). On the protein level, *Tc-*CRLF3 shares 28% sequence similarity with its human and mouse orthologs, 29% with *X. laevis* and 26% with zebrafish (Table 2). Respective proteins of the lancelet *B. floridae* and the tunicate *C. intestinales* share 27% and 24% similarity with *Tc-*CRLF3. Analysis of phylogenetic relationships among CRLF3 proteins of these species reflects the evolutionary lineages (Figure 1B). Orthologs of mammalian (mouse and human) as well as of vertebrate (mammals together with frog and fish) CRLF3 form branches that are well-separated from the other species. The non-vertebrate species differ significantly from each other, with CRLF3 of *Tribolium* showing stronger similarity with *Ciona* than with the other chordate *Branchiostoma*.

**Table 2:** Across species similarities of amino acid sequences of cytokine receptor-like factor 3 (CRLF3) orthologs.

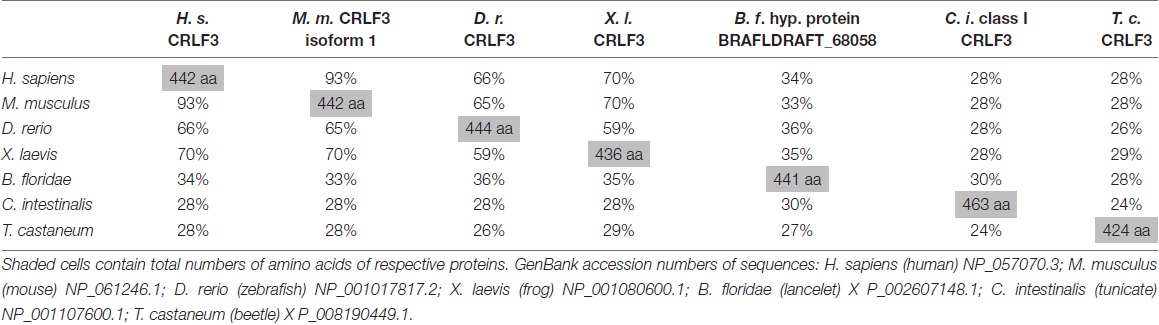

### Primary Cell Culture from Brains of *T. Castaneum*

**Figure 2:**
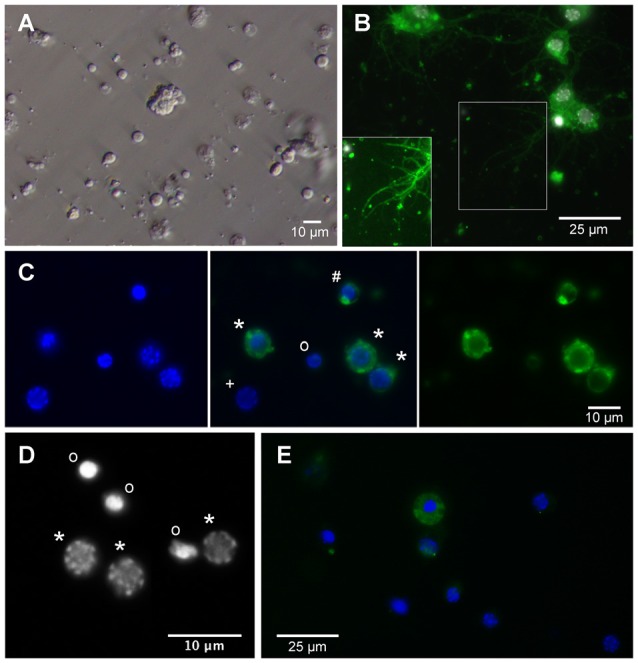
Primary cell culture from *T. castaneum* brains. **(A)** Cultured brain ells 3 h after dissociation. **(B)** Cultured brain neurons labeled with anti-horseradish peroxidase (anti-HRP) (green) and DAPI (white) after 6 days *in vitro*. Inset shows regenerated neurites with optimized brightness and contrast. **(C)** Cultured brain cells after 1 day *in vitro*. Left and middle: DAPI labeling. Right and middle: anti-HRP immunofluorescence. *Intact neurons, ^#^dying neuron, ^o^nucleus of dead or dying cell, ^+^intact glial cell. **(D)** DAPI labels heterochromatin DNA structures in nuclei of intact cells (*) and condensed nuclei with largely fragmented DNA of dead or dying cells (o). **(E)** Anti-cleaved caspase-3 immunofluorescence (green) in the cytoplasm of apoptotic cells. Dead cells (condensed DAPI-labeled nuclei) lose their cleaved caspase-3 associated fluorescence after disintegration of cellular membranes. Activated caspase-3 is absent from the cytoplasm of intact cells with discontinuously DAPI-labeled nuclei.

Freshly dissociated primary cell cultures contained round shaped cells that were typically devoid of processes (Figure 2A). Neurons and eventually also glia lose their axons, dendrites and other processes during the dissociation procedure. It is assumed that a large portion of cells is irreversibly damaged during this process preventing their attachment to the concanavalin A-coated cover slips and subsequent survival *in vitro*. After 1 day, cultures in full medium with 5% FBS contained approximately 65% alive cells while survival in serum-free cultures was reduced to approximately 40% of adherent cells. Primary cultured neurons regenerated neurites that became visible after 3 days *in vitro* and subsequently increased in length and complexity of arborizations (Figure 2B). Whether anatomical contacts of processes developed into functional synapses was not studied. Neurons and glia were distinguished by the presence or absence of anti-HRP immunoreactivity (Figure 2C). Anti-HRP has been demonstrated to label neuron-specific membrane proteins in various arthropods and anti-HRP immunocytochemistry is a simple, reliable and widely used method to identify insect neurons (Jan and Jan, 1982; Sun and Salvaterra, 1995; Gocht et al., 2009). However, since cytoplasmic membranes disintegrate in dying or dead cells, anti-HRP immunocytochemistry cannot distinguish between dead neurons and glia (e.g., the nucleus marked with “o” in the center of Figure 2C). As it was previously described for primary cultures from locust brains (Vanhems and Delbos, 1987; Gocht et al., 2009), fresh cultures from *T. castaneum* brains contained low numbers of glia and no glial cells were detected after 4 days *in vitro*. Viability of primary cultured cells was reflected in the pattern of the DNA-binding fluorescent dye DAPI (Figure 2D). Nuclei of intact cells (both neurons and glia, Figure 2C) display a patchy staining pattern that corresponds to intact chromatin structures. DNA fragmentation, that goes along with apoptotic cell death, leads to condensed and uniformly DAPI-labeled nuclei. Apoptotic cell death in cultured insect neurons can be induced by serum-deprivation, hypoxia and other physiological stressors (Jenkins et al., 2013; Miljus et al., 2014). Caspase activation involved in apoptosis can be detected in *T. castaneum* brain cell cultures by anti-cleaved caspase-3 immunocytochemistry (Figure 2E).

### Epo and EV-3 Increase the Survival of *T. Castaneum* Brain Neurons

**Figure 3:**
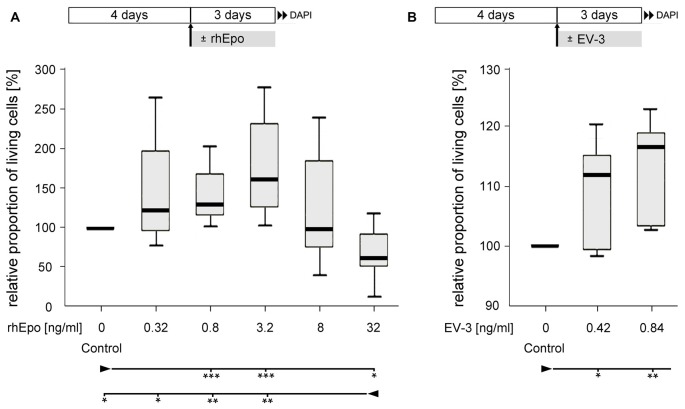
Effect of recombinant human Epo (rhEpo) and EV-3 on cellular survival of *T. castaneum* primary brain cell cultures after 7 days *in vitro*. **(A)** In comparison to untreated control cultures 0.8 and 3.2 ng/ml rhEpo significantly increase cellular survival (*p* = 0.00088 for both). In contrast, 32 ng/ml rhEpo decreases cellular survival compared with untreated control cultures (*p* = 0.01202) and cultures exposed to lower concentrations of rhEpo (0.32 ng/ml: *p* = 0.02603, 0.8 ng/ml: *p* = 0.00233, 3.2 ng/ml: *p* = 0.00233). *N* = 8, 64,518 cells evaluated. **(B)** EV-3 increases cellular survival (0.42 ng/ml: *p* = 0.04762; 0.84 ng/ml: *p* = 0.00794). *N* = 7, 42,355 cells evaluated Statistics: non-parametric Kruskal-Wallis test and Mann-Whitney *U* test with Benjamini-Hochberg correction. ***p < 0.001, **p < 0.01, *p < 0.05. Significance is shown with respect to the treatment group marked by the arrowhead. Schematics in the upper part describe experimental procedure with initial 4 days of culturing in full medium and 3 days of rhEpo or EV-3 exposure.

Cellular survival in untreated control cultures and cultures exposed to different concentrations of rhEpo or EV-3 was assessed after 7 days *in vitro*. Pharmacological treatment started after 4 days *in vitro*, when cultures were devoid of glia and exclusively contained neurons that could be labeled with anti-HRP immunocytochemistry (Figures 2B,C). rhEpo concentrations of 0.8 and 3.2 ng/ml significantly increased neuronal survival in cultures deriving from identical pools of brain cells (both with *p* < 0.001 compared to controls) while 32 ng/ml rhEpo reduced viability (*p* < 0.05 compared to control; Figure 3A). The human non-erythropoietic Epo splice variant EV-3 also significantly (*p* < 0.05 for 0.42 ng/ml EV-3 and *p* < 0.01 for 0.84 ng/ml EV-3 compared to control) increased the survival of *T. castaneum* brain neurons *in vitro* (Figure 3B).

### RNAi in Cultured Brain Neurons

**Figure 4:**
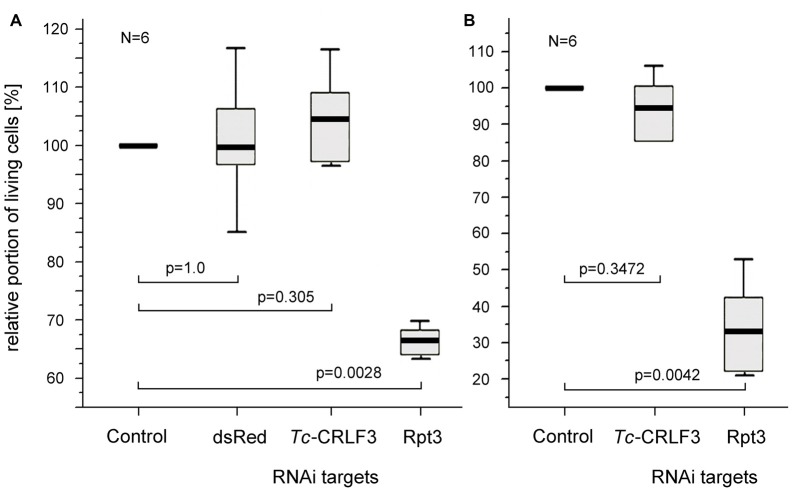
Soaking RNAi in primary cultures of *T. castaneum* brain cells. **(A)** Cellular survival after 4 days in culture medium without serum (control) and supplemented with 10 ng/μl double stranded RNA (dsRNA) to suppress expression of the anemonal protein dsRed that is not naturally expressed in insects, *Tc-*CRLF3 (fragment 2) and the proteasomal protein Rpt3. *N* = 6, 60,498 cells evaluated. **(B)** Cellular survival after 4 days in culture medium without serum (control) and supplemented with 10 ng/μl dsRNA to suppress expression of *Tc-*CRLF3 (fragment 1) and the proteasomal protein Rpt3. *N* = 6, 66,505 cells evaluated. Proportions of surviving cells were normalized to control cultures from the same pool of brain cells without dsRNA supplement (=100%). Statistics: non-parametric Kruskal-Wallis test and Mann-Whitney *U* test with Benjamini-Hochberg correction.

RNAi by application of dsRNA to the extracellular space is frequently described as soaking RNAi. In order to test, whether the mechanisms underlying dsRNA uptake and RNAi are retained in cultured brain cells, dsRNA fragments targeting the expression of three different proteins (one that does not exist in *T. castaneum*, one that is essential for cellular survival and two fragments targeting *Tc-*CRLF3) were applied to the culture medium for 4 days. DsRNA targeting the expression of Rpt3 dramatically reduced cellular survival in comparison to untreated control cultures that derived from the same pools of brains (Figures 4A,B), indicating effective dsRNA uptake and knockdown of expression of this essential proteasomal protein. In contrast, cell survival was not affected by dsRNA targeting dsRed, a protein that is not naturally expressed in insect cells (Figure 4A). Thus, uptake of dsRNA *per se* did not compromise the viability of cultured brain cells. Similarly, dsRNA targeting the expression of *Tc-*CRLF3 (fragment 2: Figure 4A and fragment 1: Figure 4B) had no impact on cellular survival, suggesting that the reduction of *Tc-*CRLF3 expression is not critical for cell survival under normal culture conditions.

### Knockdown of *Tc*-CRLF3 Expression Abolishes the Neuroprotective Effect of rhEpo

**Figure 5:**
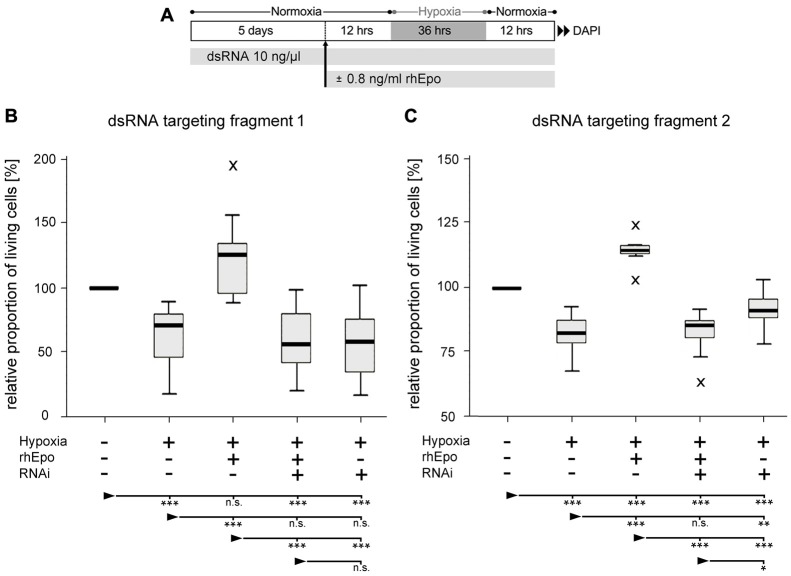
Downregulation of CRLF3 expression abolishes rhEpo-mediated neuroprotection of hypoxia-exposed neurons. **(A)** Schematic representation of experimental procedure. **(B)** RNAi with dsRNA fragment 1 of the *Tc-*CRLF3 transcript. *N* = 16, 127,808 cells evaluated. **(C)** RNAi with dsRNA targeting fragment 2 of the *Tc*-CRLF3 transcript. *N* = 14, 134,200 cells evaluated. Statistics: non-parametric Kruskal-Wallis test and Mann-Whitney *U* test for unmatched samples. *P* values were corrected with the Benjamini-Hochberg procedure. ***p < 0.001, **p < 0.01, *p < 0.05, n.s. not significant. Significance is shown with respect to the treatment group marked by the arrowhead.

In order to evaluate the role of *Tc-*CRLF3 in Epo-mediated neuroprotection, we performed two independent series of experiments (experimental procedure shown in Figure 5A) in which *Tc-*CRLF3 expression was suppressed by interference with two different dsRNAs, targeting different, non-overlapping fragments of the transcript (fragment 1 and fragment 2). Exposure to hypoxia for 36 h significantly reduced cellular survival in comparison to cultures that were maintained under normoxic conditions (Figure 5B, *p* < 0.001; Figure 5C, *p* < 0.001). Incubation with 0.8 ng/ml rhEpo completely prevented hypoxia-induced cell death (Figure 5B, *p* < 0.001; Figure 5C, *p* < 0.001). Since all cultures were maintained in serum-free medium, which acts as a mild stressor for cellular survival, survival in rhEpo-treated/hypoxia-exposed cultures was even higher than in normoxic controls. RNAi-mediated knockdown of *Tc-*CRLF3 expression completely abolished the neuroprotective effect of rhEpo in hypoxia-exposed neuronal cultures. RNAi targeting both fragment 1 (Figure 5B) and fragment 2 (Figure 5C) significantly decreased cellular survival compared to rhEpo-treated/hypoxia-exposed cultures (both with *p* < 0.001) and normoxic controls (both with *p* < 0.001). *Tc*-CRLF3-directed RNAi had no additional deleterious effect on hypoxia-exposed neuronal cultures (Figures 5B,C) which is in line with our control experiment where *Tc-crlf3* RNAi did not change survival in normal cell cultures (Figure 4). Thus, knockdown of *Tc-*CRLF3 expression eliminates Epo-mediated neuroprotection of *T. castaneum* brain neurons, without compromising cellular survival under neither normoxic (Figures 4A,B) nor hypoxic (Figures 5B,C) conditions. This confirms the assumption that *Tc-CRLF3* is the receptor mediating Epo neuroprotective effects in *T. castaneum*.

## Discussion

Cytokines and their receptors coordinate many physiological processes that adapt organisms to changing environments and maintain cellular functionality under unfavorable conditions. Cytokines include a heterogeneous collection of glycoproteins that typically activate multiple types of receptors. Cytokine receptors, typically hetero- or homo-multimeric complexes, can be activated by multiple different cytokine ligands (review: Dinarello, 2007). Overlapping and complementary functions of cytokines together with low primary sequence similarities of cytokines and many types of cytokine receptors complicate across-species comparison and studies on their evolutionary origins (Beschin et al., 2001; Liongue and Ward, 2007). As an exception, CRLF3 is phylogenetically well-conserved and orthologs were detected in mammals, frog, fish, non-vertebrate chordates and some, but not all insects (this study; Wyder et al., 2007). CRLF3 has been classified as a type I cytokine receptor of group 1 (“prototypic type I cytokine receptors”) that also includes thrombopoietin receptor, prolactin receptor, growth hormone receptor and classical EpoR (Boulay et al., 2003). Cytokine receptors of this group are single pass transmembrane receptors that associate to mono- or heteromeric complexes and transduce their activation into the cell through interaction with JAKs.

We established adherent primary cell cultures from brains of *T. castaneum*. Culture conditions supported the survival of neurons and eliminated glial cells within 4 days *in vitro*. Similar observations were previously made with primary brain cell cultures form *L. migratoria* (Gocht et al., 2009). Successful RNAi-mediated downregulation of protein expression has previously been demonstrated in various developmental stages of *T. castaneum* (Bucher et al., 2002; Ulrich et al., 2015). In this study we extended the application of RNAi to *T. castaneum*-derived neuronal cell cultures, by showing that expression of particular proteins (such as rpt3 and *Tc*-CRLF3) can be suppressed by soaking dsRNAi.

Epo promoted the survival of primary neuron cultures from *T. castaneum* brains and effectively suppressed apoptotic cell death induced by hypoxia and serum deprivation. This confirmed the results from previous studies with primary neuronal cultures from locust brains (Ostrowski et al., 2011; Miljus et al., 2014) and suggested that Epo-mediated neuroprotection might be a common mechanism in those insects that contain orthologs of CRLF3 (e.g., *T. castaneum*, *L. migratoria*, *G. bimaculatus* but not *D. melanogaster*). RNAi-mediated knockdown of *Tc-*CRLF3 expression completely abolished the neuroprotective effect of Epo, indicating that this cytokine receptor is essential for initiating anti-apoptotic mechanisms in *T. castaneum* brain neurons. In order to minimize the risk that suppressed expression of another protein besides *Tc-*CRLF3 interferes with Epo-mediated neuroprotection, *Tc-CRLF3* expression was knocked down with two different dsRNA constructs that targeted different, entirely non-overlapping sequences of the *Tc-crlf3* gene transcript. Both dsRNA constructs abolished Epo-mediated protection of hypoxia-exposed beetle neurons. Complete loss of Epo-mediated neuroprotection was previously achieved by co-incubating hypoxia-exposed locust brain neurons with JAK and STAT inhibitors (Miljus et al., 2014), suggesting that *Tc-*CRLF3 is an upstream component of this neuroprotective transduction pathway. This is in line with the presence of predicted JAK docking sites in *T. castaneum* and human CRLF3 (this study; Boulay et al., 2003) and the observation that human CRLF3 activates STAT3 (Yang et al., 2009). However, *Tc-*CRLF3-directed RNAi did not compromise neuronal survival in normoxic and hypoxia-exposed neuronal cultures, suggesting that it is not essential for cellular survival, though being indispensable for Epo-mediated neuroprotection. Similarly, studies on PC12 and HEK cells have not reported any obvious decrease in viability following CRLF3 knockdown (Yang et al., 2009; Hashimoto et al., 2012).

Epo-mediated neuroprotection shares a number of common mechanisms between mammals (mostly studied in rodents) and insects (studied in locusts and beetles). In both groups of animals neuroprotection is mediated via JAK and STAT activation, while mammalian neurons may additionally employ transduction pathways involving MAPK, PI3K and NF_κ_B (Digicaylioglu and Lipton, 2001; Sirén et al., 2001; Miljus et al., 2014). Various studies demonstrated optimum-type dose-response curves for different concentrations of experimentally applied Epo (this study; Sirén et al., 2001; Weishaupt et al., 2004; Ostrowski et al., 2011; Miller et al., 2015) and Epo-induced receptor endocytosis has been described for locust neurons (Miljus et al., 2017) and mammalian erythroid progenitors (Bulut et al., 2011). Importantly, the non-erythropoietic splice variant EV-3 increased the survival of serum-deprived primary cultured *T. castaneum* brain neurons (this study) and protected both hypoxia-exposed locust brain neurons (Miljus et al., 2017) and rat cortex neurons challenged with glucose and oxygen deprivation (Bonnas, 2012; Bonnas et al., 2017) from apoptotic cell death. This suggested a greater similarity of the ligand binding regions of locust and mammalian neuroprotective Epo receptors than between mammalian neuroprotective and classical erythropoietic EpoR.

CRLF3 has been implicated with typical cytokine-regulated processes such as cell cycling and cellular morphogenesis (Yang et al., 2009; Hashimoto et al., 2012) and alterations of its expression have been detected in various types of tumors (Dang et al., 2006; Yang et al., 2009) and patients affected by ALS (Cirulli et al., 2015). However, the detailed cellular function of CRLF3 has not been resolved and descriptions of its functional domains and its subcellular localization are inconsistent. The present study deorphanizes CRLF3 by identifying Epo-like signaling molecules as its ligand. Preliminary own studies detected CRLF3 expression in mouse brain, liver and kidney, suggesting that it mediates functions in large parts of mammalian organisms. Whether it functions as a neuroprotective or, more general, as a cytoprotective Epo receptor in mammals including humans needs to be studied by appropriate assays. Insects, such as *T. castaneum*, but not *Drosophila melanogaster* that lack both a CRLF3 ortholog and Epo-mediated neuroprotection, may be used to search for additional tissue-protective Epo receptors with conserved orthologs. Since insects lack erythrocytes and “classical” EpoR, studies do not have to consider interference of these mechanisms. Moreover, as insects do not contain *epo* genes that can be identified by sequence similarity, an “alternative” ligand awaits to be identified that activates insect neuroprotective receptors such as CRLF3 and may have mammalian orthologs with similar functions.

## Author Contributions

NH, DYK, JL, KP, MB and RH performed experiments and analyzed data. NH, LW, MB, GB, HE and RH designed the study and wrote the manuscript.

## Conflict of Interest Statement

The authors declare that the research was conducted in the absence of any commercial or financial relationships that could be construed as a potential conflict of interest.

## Footnotes

^1^http://asgard.rc.fas.harvard.edu/blast.html

## Supplementary Material

FIGURE S1 5′ to 3′ strand of double stranded RNA (dsRNA) fragments used for RNAi in primary brain cell cultures from *T. castaneum*.

FIGURE S2 Aligned amino acid sequences of human cytokine receptor-like factor 3 (CRLF3; NCBI GenBank NP_057070.3) and orthologous *au3.g2971.t1*-derived protein *Tc*-CRLF3 from *T.castaneum*. Predicted domains are indicated by bars on top of the sequence. Domain information related to human CRLF3 derived from NCBI (http://www.ncbi.nlm.nih.gov/protein/Q8IUI8.2) and (24).
