# Supplementary figures and images for: The Insect Ortholog of the Human Orphan Cytokine Receptor CRLF3 Is a Neuroprotective Erythropoietin Receptor
Source: Front Mol Neurosci. 2017 Jul 14;10:223. doi: 10.3389/fnmol.2017.00223 (PMC5509957; doi:10.3389/fnmol.2017.00223)

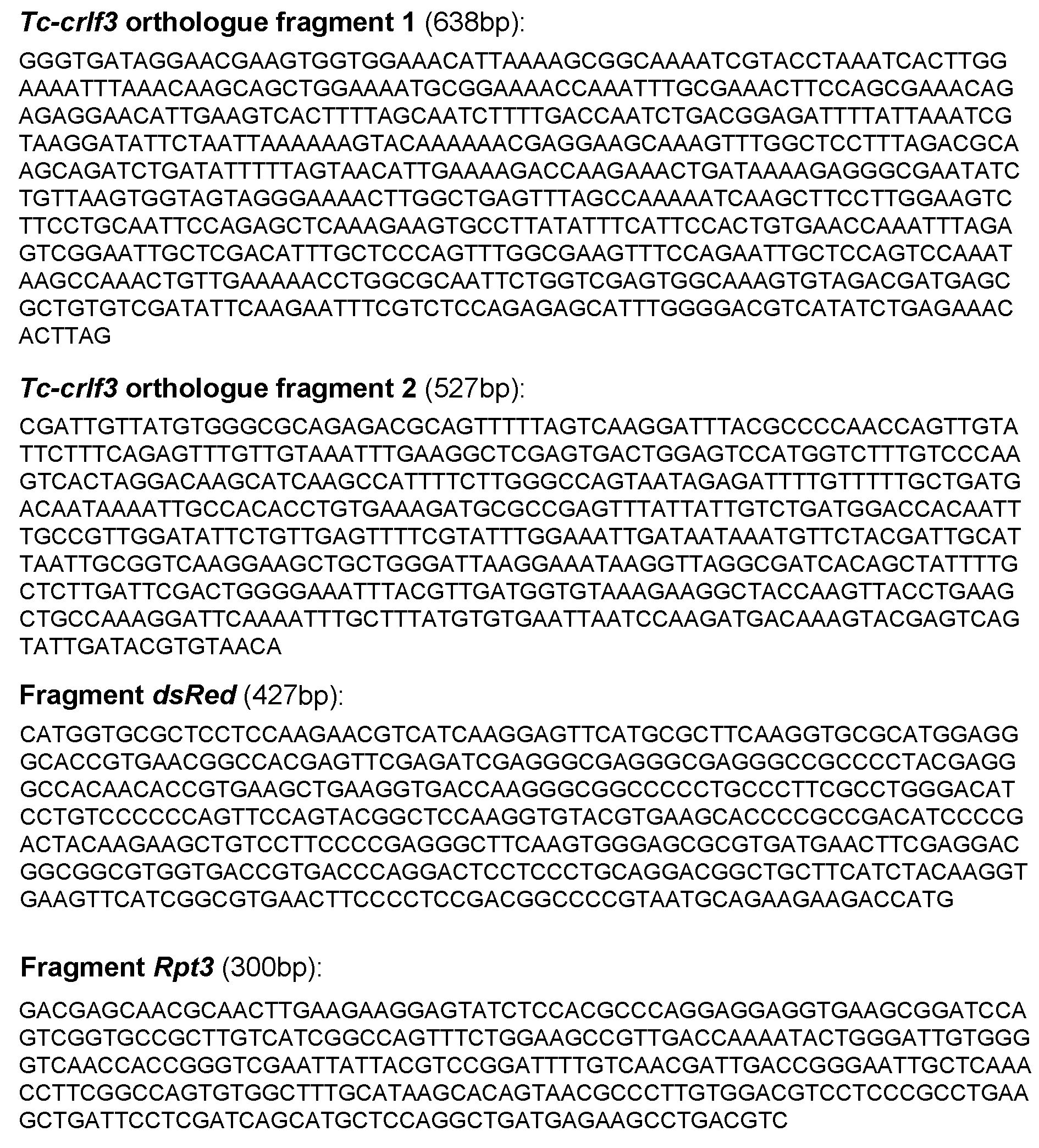

Supplement: FIGURE S1 — 5′ to 3′ strand of double stranded RNA (dsRNA) fragments used for RNAi in primary brain cell cultures from T. castaneum. [file Image_1.tif]

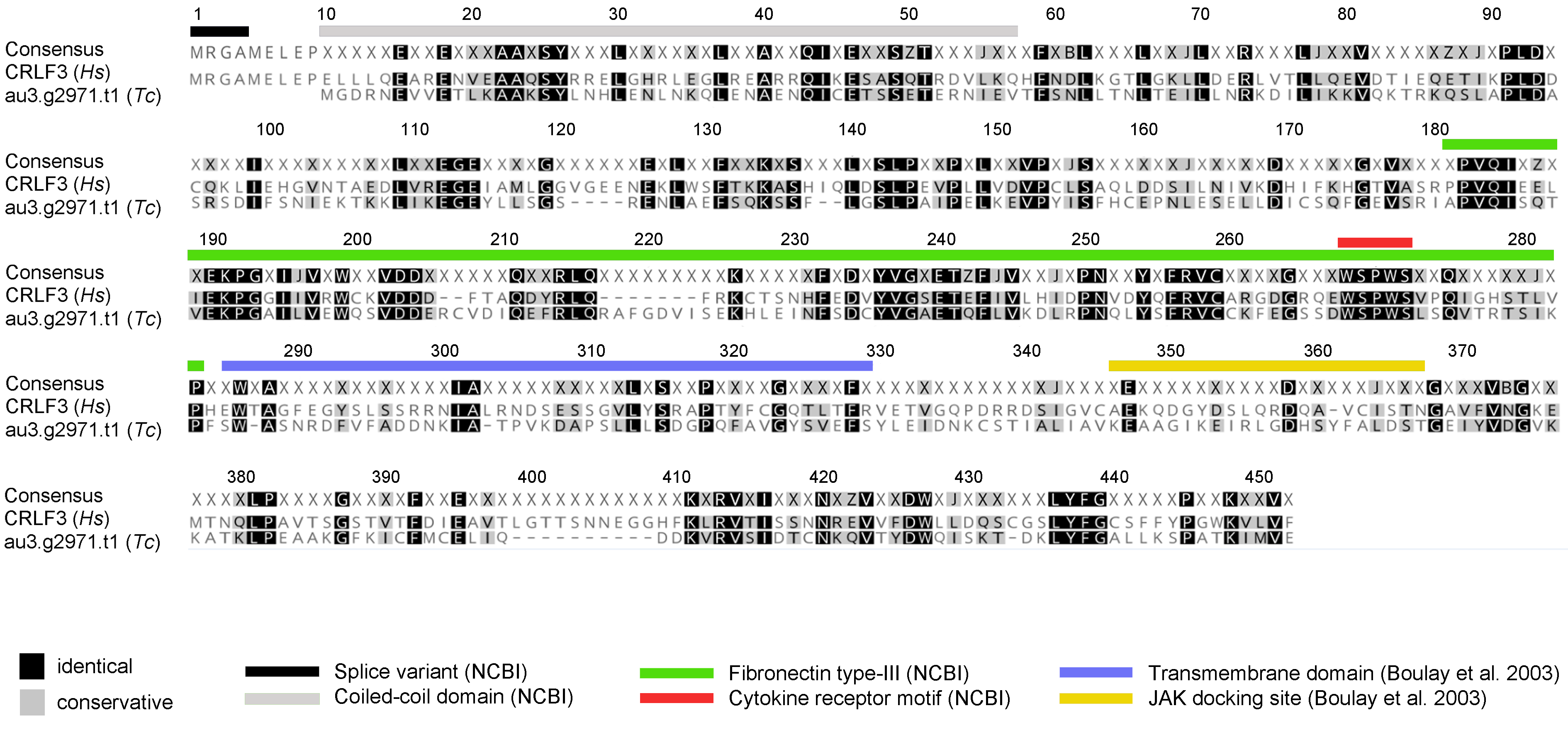

Supplement: FIGURE S2 — Aligned amino acid sequences of human cytokine receptor-like factor 3 (CRLF3; NCBI GenBank NP_057070.3) and orthologous au3.g2971.t1-derived protein Tc-CRLF3 from T.castaneum. Predicted domains are indicated by bars on top of the sequence. Domain information related to human CRLF3 derived from NCBI (http://www.ncbi.nlm.nih.gov/protein/Q8IUI8.2) and (24). [file Image_2.tif]
